# Earth-friendly micellar UPLC technique for determination of four hypoglycemic drugs in different pharmaceutical dosage forms and spiked human plasma

**DOI:** 10.1186/s13065-023-00983-6

**Published:** 2023-07-12

**Authors:** Manal S. Elmasry, Wafaa S. Hassan, Hanan A. Merey, Israa M. Nour

**Affiliations:** 1 Analytical Chemistry Department, Faculty of Pharmacy, Zagazig University, Zagazig, 44519 Egypt; 2 Analytical Chemistry Department, Faculty of Pharmacy, Cairo University, Kasr-El-Aini, Cairo, 11562 Egypt; 3 Analytical Chemistry Department, Faculty of Pharmacy, October 6 University, 6 October City, Giza, 12585 Egypt; 4 Pharmaceutical Chemistry Department, Faculty of Pharmacy, Egyptian Russian University, Badr, 11829 Egypt

**Keywords:** Micellar UPLC, Spiked human plasma, Pioglitazone, Alogliptin, Glimepiride, Vildagliptin.

## Abstract

A novel, sensitive, and green micellar UPLC method was proposed and validated for the simultaneous determination of four hypoglycemic agents used in type II diabetes mellitus treatment namely, pioglitazone, alogliptin, glimepiride, and vildagliptin. The developed UPLC method was successfully applied for quantitative analysis of these drugs in bulk, in pharmaceutical formulations, and in spiked human plasma. Chromatographic separation was carried out on a Kinetex® 1.7 μm XB-C18 100 Å (50 × 2.1 mm) column, using a degassed and filtered mixture of (0.1 M SDS- 0.3% triethyl amine- 0.1% phosphoric acid (pH 6)) and n-propanol (85:15 v/v), at a flow rate of 0.2 mL/min. The experimental conditions of the suggested method were well investigated and optimized. The newly developed micellar UPLC method is capable of determining different dosage forms at the same time with the same solvents, saving time and effort. The method was found to be efficiently applicable in spiked human plasma and could be extended to study the pharmacokinetics of the cited drugs in real human plasma samples. The greenness of the developed method was evaluated by applying the Eco-scale scoring tool, which verified the excellent greenness of the analytical method.

**Supplementary Information:**

The online version contains supplementary material available at 10.1186/s13065-023-00983-6.

## Introduction

Type 2 diabetes mellitus (T2DM) is a chronic metabolic syndrome that resulted from insulin deficiency which leads to hyperglycemia and serious complications such as micro- and macrovascular damage [1]. Management of T2DM can be achieved via treatment with oral hypoglycemic medications and by encouraging patients to change their lifestyle by following a balanced diet and regular exercise to control their blood glucose levels [2]. Oral hypoglycemic drugs can be used safely to control the blood glucose level in T2DM patients by different modes of action but due to the progressive characteristic of T2DM, a combination of oral hypoglycemic agents therapy is required [3].

**Fig. 1:**
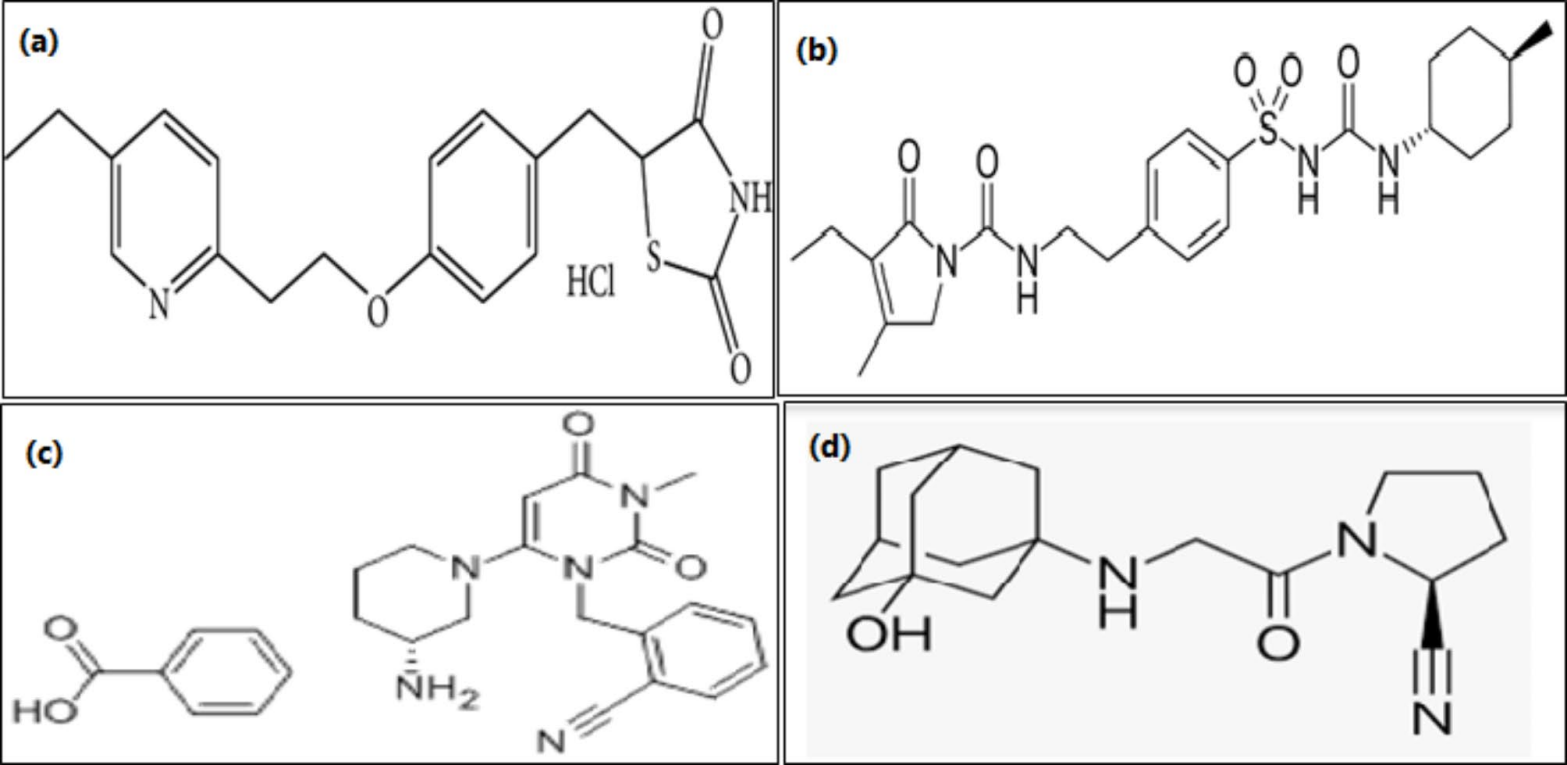
Chemical structure of (**a**) Pioglitazone, (**b**) Glimepiride, (**c**) Alogliptin, and (**d**) Vildagliptin

Pioglitazone (PIO) Fig. 1a, 5-[[4-[2-(5-ethylpyridin-2-yl) ethoxy] phenyl]methyl]-1, 3-thiazolidine-2, 4-dione [4] is from thiazolidinediones that are indicated for the treatment of T2DM as it improves insulin sensitivity and increases glucose uptake [5]. Glimepiride (GLM) Fig. 1b, 1-[[p-[2-(3-ethyl-4-methyl-2-oxo-3-pyrroline-1-carboxamido) ethyl] phenyl] sulfonyl]-3-(trans-4-methylcyclohexyl) urea [4], is a third-generation sulfonylurea with hypoglycemic activity, it increases the secretion of insulin by activating beta cells and is used for the management of T2DM to improve blood glucose control [6]. Alogliptin (ALO) Fig. 1c, 2-[[6-[(3R)-3-aminopiperidin-1-yl]-3-methyl-2,4-dioxopyrimidin-1- yl]methyl]benzonitrile [7] and vildagliptin (VLD) Fig. 1d, (2 S)-1-[2-[(3-hydroxy-1-adamantyl) amino] acetyl] pyrrolidine-2-carbonitrile [4] are Dipeptidyl Peptidase-4 (DPP-4) inhibitors that are used in the therapy of T2DM via increasing Insulin secretion and suppressing glucagon secretion [8].

Many clinical studies have proven that some combination therapy of hypoglycemic agents in T2DM patients can exert a superior blood glucose control than mono or single-ingredient therapy [9]. Pioglitazone (PIO) and Alogliptin (ALO) are present in the market as a combined therapy that is used for the management of T2DM under the trade name Prandaglim plus (15 mg pioglitazone/ 25 mg alogliptin). It was found that the combination of ALO and PIO gives a better effect in the treatment of T2DM than the effect of single drug treatment by improving the function of beta-cells [10]. The literature review reported a few analytical techniques for the estimation of PIO and ALO including spectrophotometric methods (area under the curve, first derivative of ratio spectra, and second order derivative) [11, 12], HPTLC [13, 14], LC-MS/MS in human plasma [15, 16] and RP-HPLC [17–19].

Pioglitazone (PIO) and Glimepiride (GLM) are available in the market under the trade name Zanoglide (30 mg pioglitazone / 4 mg glimepiride) combined therapy that is used for the treatment of T2DM patients who have a cardiovascular risk. Studies demonstrated that the combination treatment of pioglitazone with glimepiride can have a dual action of glycemic control and improving the body’s lipid profile and lowering cardiovascular risk [20]. Literature data reported a fewer number of analytical methods for quantitative analysis of PIO and GLM involving spectrophotometric methods (oxidative coupling and chemometric techniques) [21, 22], native spectrofluorimetry [23], LC/ tandem mass in human plasma [24] and RP-HPLC [25–29].

Vildagliptin (VLD) can be added to Glimepiride (GLM) for the therapy of T2DM. It is effective in improving glycemic control and well tolerated in diabetic patients when added to glimepiride as it decreases the risk of hypoglycemia and gaining weight [30, 31]. Some studies demonstrate that adding vildagliptin (VLD) to Pioglitazone (PIO) was observed to be more dynamic in glycemic control by maintaining *β*-cell function and lowering insulin resistance than either monotherapy component [32].

Literature data reports a few chromatographic methods for the simultaneous determination of VLD with GLM or PIO in bulk and commercially available drug products [33, 34]. Interestingly, none of the previous methods reported the simultaneous estimation of the studied drugs.

A Straightforward and well-established branch of high-performance liquid chromatography is micellar liquid chromatography (MLC). MLC has been used frequently to identify various substances in pharmaceutical medications [35, 36], biological fluids [37, 38], and food products [39].

The MLC method has multiple advantages over other conventional HPLC methods as MLC would enable concurrent separation of hydrophilic and hydrophobic solutes in the same run. MLC has rapid gradient capability, distinct separation selectivity, excellent reproducibility, reliability, robustness, improved detection, and affordability. MLC is an excellent ecological alternative for conventional HPLC since it enhances both environmental and economic influences. Also, the capacity of micellar mobile phase to dissolve sample proteins makes direct sample injection of biological samples into the column one of the principal applications of MLC [40].

In this work, a new and sensitive micellar UPLC method (MUPLC) was developed for the determination of PIO, ALO, GLM, and VLD in pure form, dosage forms, and spiked human plasma. Compared to the reported methods, the newly developed MUPLC method has advantages including better sensitivity, economic, low consumption of organic solvent, enhancement of the resolution, and eco-friendly to the environment.

## Experimental

### Material and reagents

Pure standards of ALO (99.85%), PIO (100.87%), GLM (99.32%), and VLD (99.91%) were kindly provided by EVA Pharma (Cairo, Egypt). Zanoglide 4/30 tablet, Batch number 2,011,283, labeled to contain 4 mg GLM and 30 mg PIO per tablet. Prandaglim plus 25/15 tablet, Batch number 2,103,041, labeled to contain 25 mg ALO and 15 mg PIO per tablet. Prandaglim 25 mg tablet, Batch number 2,001,171, each tablet contains 25 mg ALO as labeled. Gliptus 50 mg tablet, Batch number 2,010,622, labeled to contain 50 mg VLD. All tablets were manufactured by EVA Pharma (Cairo, Egypt) and purchased from the local market. Sodium dodecyl sulphate (SDS) is provided from (Merk, Germany).

Analytical grade orthophosphoric acid and triethyl amine and HPLC grade acetonitrile and n-propanol were purchased from Sigma-Aldrich (Germany). De-ionized water was freshly obtained in-house by the Millipore water purification system. Plasma samples were obtained from Al-Azhar University Hospital in Cairo, Egypt, and kept refrigerated till assayed.

### Apparatus

The HPLC chromatographic system consisted of Agilent (1100 series) equipped with a quaternary pump (G 1311 A), and an automatic injector equipped with a 1 µl sample loop injector. Detection was achieved by UV-detector (model G1314 A). UPLC Core-Shell column Kinetex® 1.7 μm XB-C18 100 Å (50 × 2.1 mm) (USA). The mobile phase was degassed by a degasser (model G1322A). Jenway pH–Meter was purchased from the UK. Analytical balance (Precisa125A, Switzerland). Vortex (model IVM-300P, Taiwan), Benchtop centrifuge with (Hunan, China).

### Chromatographic condition

- **Stationary phase**: Kinetex® 1.7 μm XB-C18 100 Å (50 × 2.1 mm) column, the column temperature was ambient, and the injection volume was 1 µl.
- **Mobile phase**: the mobile phase that was the best for chromatographic separation was consisting of solvent A: solvent B (85:15 v/v).
  - **Solvent A**: degassed and filtered mixture of [0.1 M SDS- 0.3% triethyl amine- 0.1% phosphoric acid (pH 6)].
  - **Solvent B**: n-propanol.
- **Flow rate**: 0.2 mL/min.
- **Detection**: UV detection at 225 nm.

### Standard solutions

An accurately weighed amount of 10 mg of PIO, ALO, GLM, and VLD was transferred into a 100 ml volumetric flask and dissolved in 50 mL of the mobile phase then completed to volume with the same mobile phase to obtain a 100 µg/mL stock solution of each drug.

### Procedures

#### Construction of calibration curves

Into a series of 10-mL volumetric flasks, different aliquots of PIO, ALO, GLM, and VLD were separately transferred using a micropipette from their (100 µg/mL) standard stock solutions, then the flasks were completed to volume with the mobile phase to reach a final concentration of (1–80 µg/mL) (0.1–25 µg/mL) (0.25-50 µg/mL) (0.3–50 µg/mL) for PIO, ALO, GLM, and VLD, respectively. These solutions were then transferred into vials of the autosampler and automatically injected into the column. Different calibration graphs were constructed by plotting the concentration of each drug against the corresponding peak area.

#### Procedure for pharmaceutical preparation

Five tablets of Zanoglid, Prendaglim plus, Prendaglim, and Gliptus, were separately weighed and ground in a mortar into a fine powder. Accurate weight of the powder of Zanoglid tablet (equivalent to 30 mg of PIO and 4 mg of GLM), Prendaglim plus tablet (equivalent to 15 mg of PIO and 25 mg of ALO), Prendaglim tablet (equivalent to 25 of ALO mg) and Gliptus tablet (equivalent to 50 mg of VLD) were separately transferred into 100-mL volumetric flask. 50 ml of the mobile phase was added followed by sonication for 30 min then the contents of the flasks were filtered. The flasks were completed to 100 mL volume with the same mobile phase to obtain stock solutions having a concentration of (300 µg/mL of PIO and 40 µg/mL of GLM), (150 µg/mL of PIO, and 250 µg/mL of ALO), (250 µg/mL of ALO) and (500 µg/mL of VLD). different dilutions with the mobile phase were done within the linear ranges. Separation was achieved as mentioned in the procedure section.

#### Procedure for spiked human plasma

For the calibration curve in human plasma, in a series of screw-capped tubes for centrifugation, 1 mL of human plasma was transferred into a series of centrifugation tubes and spiked with 1 mL containing different concentrations of PIO, ALO, GLM, and VLD, respectively and separately. Each centrifugation tube received 3 mL of acetonitrile (for protein denaturation), which was shaken and vortexed for 1 min. The samples were centrifuged at 4000 rpm for 30 min. The supernatants (protein-free layer) were taken and evaporated to dryness under a moderate nitrogen gas stream. The residue was dissolved in 2 ml of methanol, then transferred into a 10-mL volumetric flask and completed with methanol to the mark. 20µL of each solution was injected in triplicate using the above-mentioned chromatographic conditions, and the peak areas were calculated. The calibration curves represent the relationship between the peak areas and the corresponding concentrations in the range 5–25 µg/mL for PIO, ALO, and GLM.

#### Procedure for quality control samples

For the validation of the proposed MUPLC, three quality control samples: low (LQC), medium (MQC), and high (HQC) were prepared for the four studied drugs in concentrations of 5, 10, and 15 µg/mL. For the bioanalytical validation, low (LQC), medium (MQC), and high (HQC) samples were prepared in concentrations of 5, 15, and 25 µg/mL.

## Results and discussions

The MUPLC method was proposed for simultaneous determination of PIO, ALO, GLM, and VLD with possible application to different dosage forms including Zanoglide (PIO/GLM), Prandaglim plus (ALO /PIO), Prandaglim (ALO), Gliptus (VLD) and in spiked human plasma.

**Fig. 2:**
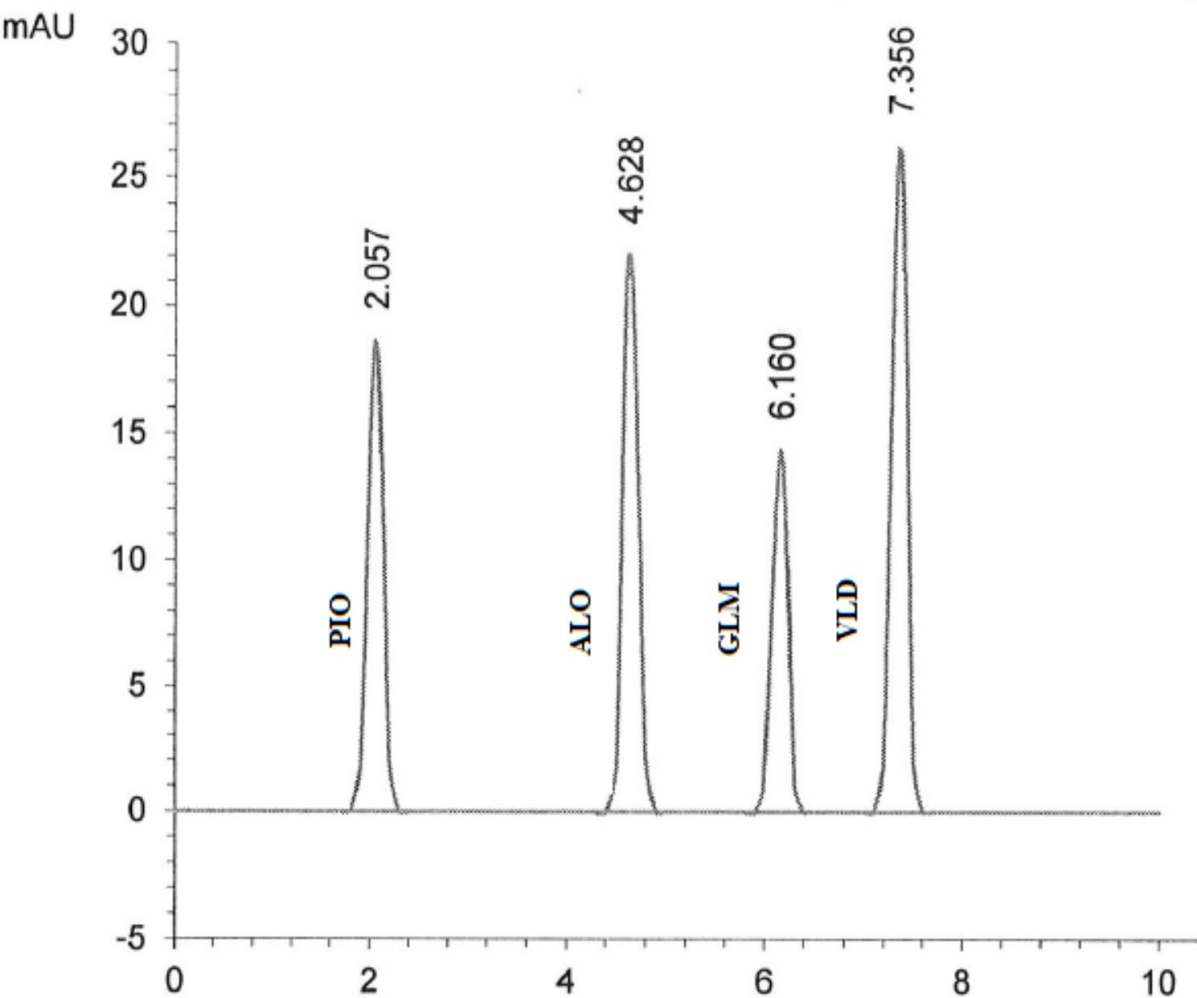
MUPLC chromatograms showing separation (20 µg/mL) of each PIO (Rt = 2.05 min), ALO (Rt = 4.62 min), GLM (Rt = 6.16 min), and VLD (Rt = 7.35 min) using the specified chromatographic conditions

**Fig. 3:**
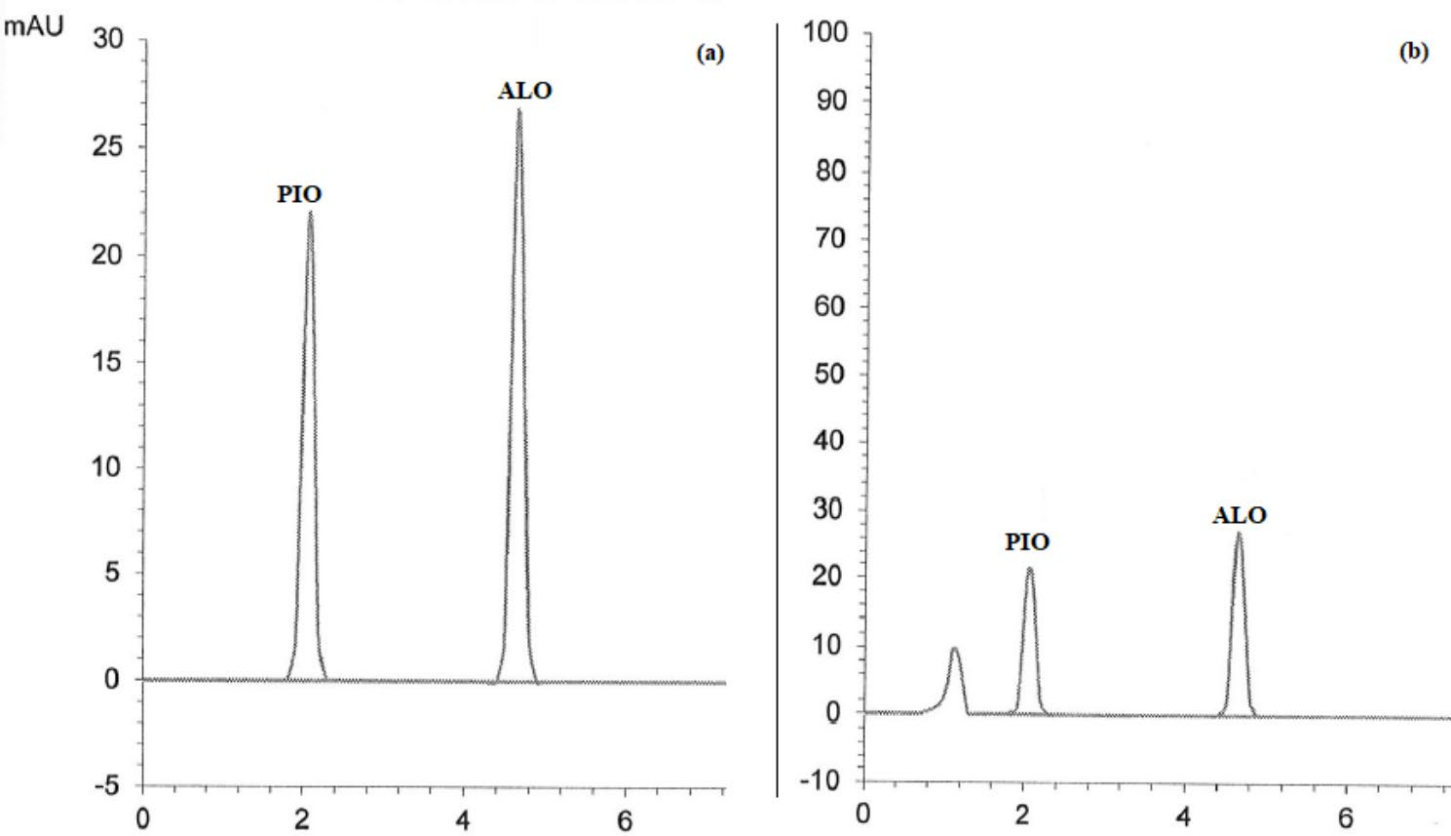
MUPLC chromatograms of PIO (Rt = 2.05 min), and ALO (Rt = 4.62 min) in (**a**) Binary mixture (25:25 µg/mL) (**b**) Spiked plasma (25:25 µg/mL)

**Fig. 4:**
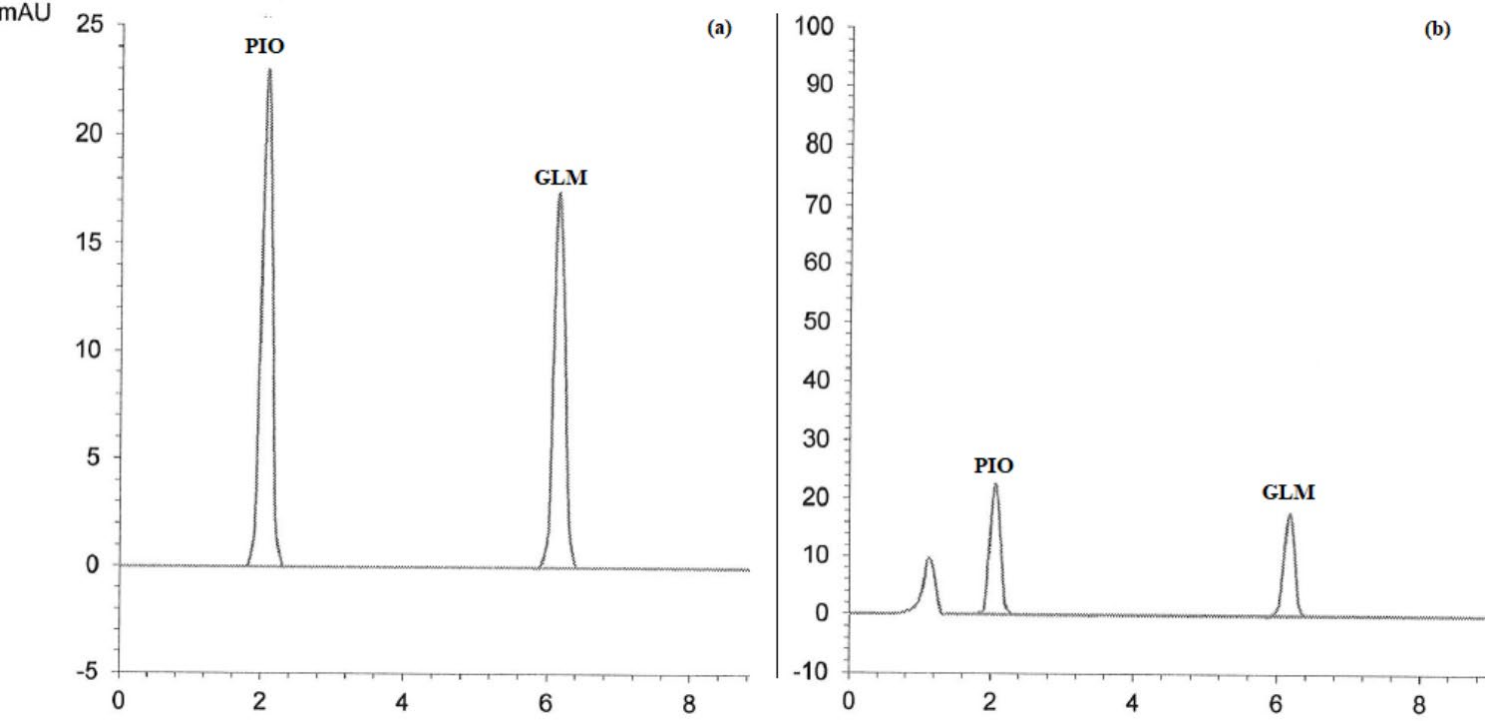
MUPLC chromatograms of PIO (Rt = 2.05 min), and GLM (Rt = 6.16 min) in (**a**) Binary mixture (25:25 µg/mL) (**b**) Spiked plasma (25:25 µg/mL)

**Fig. 5:**
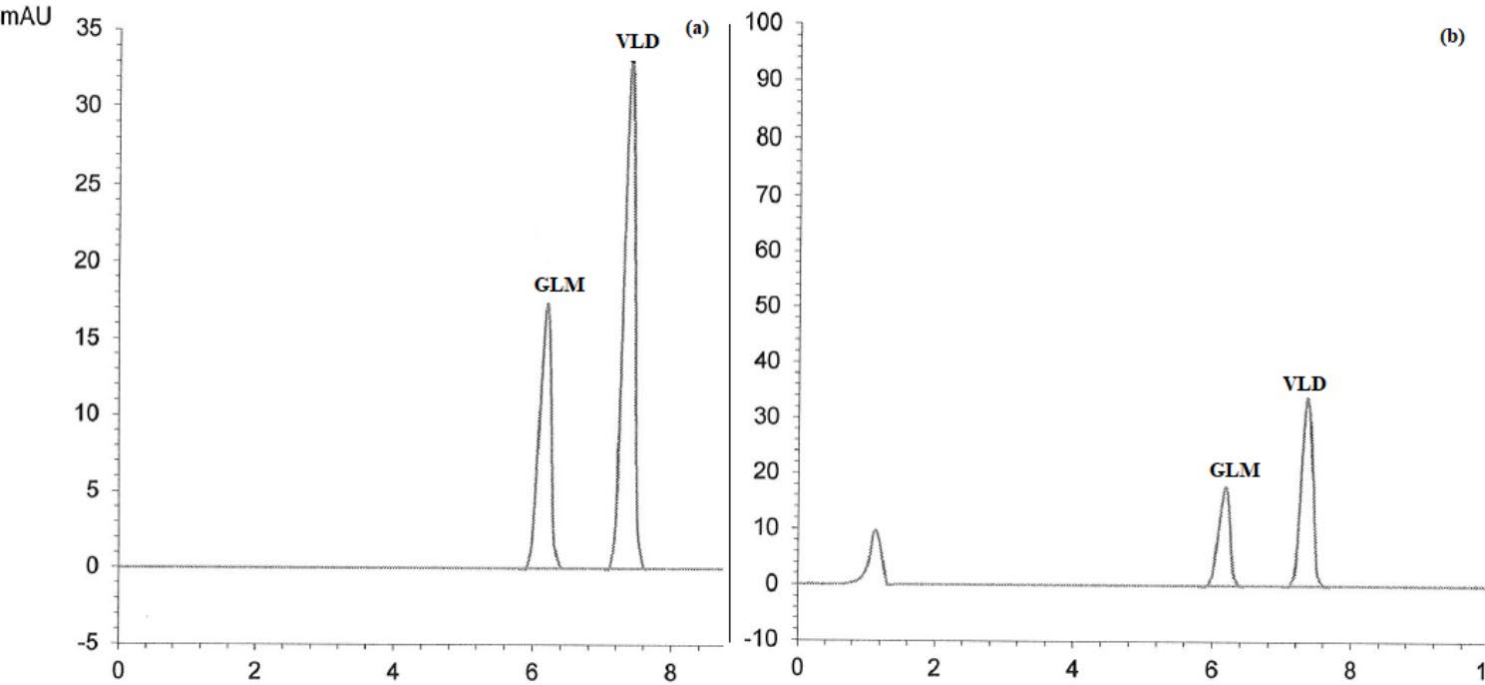
MUPLC chromatograms of GLM (R_t_ =6.16 min) and VLD (R_t_ =7.35 min) in (**a**) Binary mixture (25:25 µg/mL) (**b**) Spiked plasma (25:25 µg/mL)

**Fig. 6:**
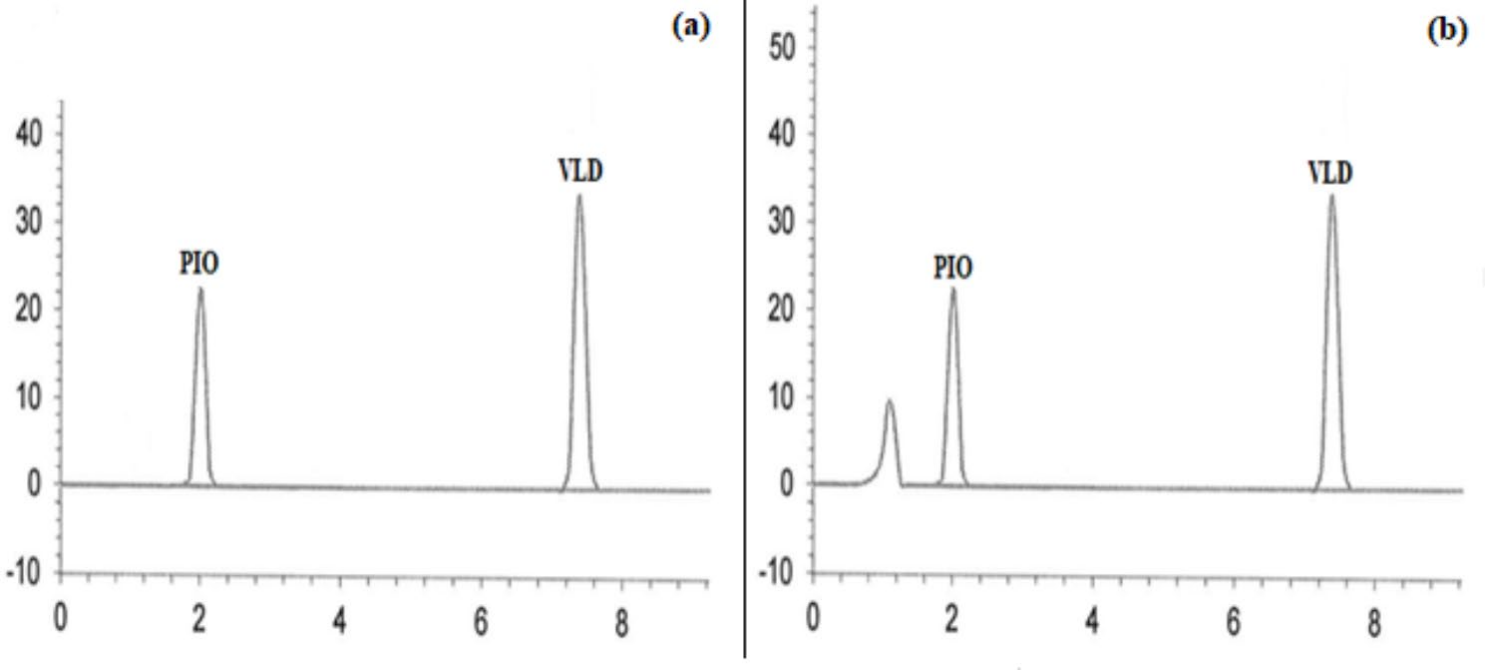
MUPLC chromatograms of PIO (R_t_ = 2.05 min), and VLD (R_t_=7.35 min) in (**a**) Binary mixture (25:25 µg/mL) (**b**) Spiked plasma (25:25 µg/mL)

Good separation of the cited drugs using the proposed MUPLC method was achieved at 2.05, 4.62, 6.16, and 7.35 min for PIO, ALO, GLM, and VLD, respectively with clear resolution between their peaks. Chromatograms in Figs. 2, 3, 4, 5 and 6 showed the separation of the studied drugs in their binary mixtures and spiked human plasma. As shown, there was no interference from the plasma matrix.

### Advantages of the proposed method

For a company that produces these drugs alone or in a mixture, this method provides easy determination of different dosage forms at the same time and in one run with the same solvents, including saving time and effort required or column washing between runs, and the reduced amount of used solvents in the mobile phase in comparison to conventional HPLC, plus the actual advantages of using the MUPLC technique that include better sensitivity, economics, low consumption of organic solvent, enhancement of the resolution, and being eco-friendly to the environment.

### Optimization of experimental conditions

In the proposed method, optimum efficient separation with fine resolution and symmetric shape peaks was achieved using a mobile phase composed of solvent A [mixture of 0.1 M SDS- 0.3% triethyl amine- 0.1% phosphoric acid (pH 6)] and solvent B (n-propanol) (85:15 v/v), on Kinetex® C18 column, with a flow rate of 0.2 mL/min and UV detection at 225 nm.

#### Choosing the optimum mobile phase composition

Different mobile phases were tried at different ratios to achieve optimum chromatographic separation. Best separation was achieved by using a mixture of [0.1 M SDS- 0.3% triethyl amine- 0.1% phosphoric acid (pH 6)] and n-propanol (85:15 v/v). the effect of changing the pH of the mobile phase was also tested and (pH 6) was the best for optimum separation. pH value was adjusted to 6 by adding 0.1% phosphoric acid for good resolution without tailing the peaks.

#### Choosing the optimum flow rate

The flow rate of the mobile phase was varied by ± 0.02 and a flow rate of 0.2 mL/ min resulted in a good resolution of peaks at a suitable retention time.

#### Choosing the optimum wavelength of detection

After trying several wavelengths from 210 to 240 nm, the UV detection of the studied hypoglycemic drugs was carried out at 225 nm at which the cited drugs showed good absorption.

### Method validation

The proposed MUPLC was validated in accordance with the ICH guidelines [41] and the bioanalytical FDA validation guidelines [42].

#### Linearity

Calibration curves were constructed relating the linear relationship between the peak area and the corresponding drug concentrations (µg/ mL) in the range of (1–80 µg/mL), (0.1–25 µg/mL), (0.25-50 µg/mL), and (0.3–50 µg/mL) for PIO, ALO, GLM, and VLD, respectively for the pure drugs. Also, good linearity for the cited drugs, with correlation coefficients of 0.9999, 0.9998, 0.9997, and 0.9991 was obtained for PIO, ALO, GLM and VLD in the 5–25 µg/mL range for the spiked human plasma. The linear range of the proposed method and the regression equations parameters were shown in Tables 1&2.

**Table 1:** Assay validation sheet of the proposed MUPLC method for the determination of PIO, ALO, GLM, and VLD.

Parameter	PIO	ALO	GLM	VLD
**Linearity range (µg/ mL)**	1–80	0.1–25	0.25-50	0.3–50
**Correlation coefficient (r** ^**)**^	0.9999	0.9999	0.9999	0.9999
**Slope**	5.6087	8.3759	4.3799	11.0076
**Intercept**	0.2519	0.1278	-0.6364	-0.3432
**SD of intercept**	0.5700	0.0839	0.0930	0.1814
**LOD (µg/ mL)**	0.3353	0.0331	0.0701	0.0544
**LOQ (µg/mL)**	1.0162	0.1002	0.2124	0.1648
**Accuracy (R %)**	101.24	98.96	99.79	100.19
**Precision**				
**Repeatability (RSD%)** ^**a**^ **Intermediate Precision (RSD%)** ^**b**^	1.424 1.822	0.965 1.068	0.552 0.997	0.987 1.256

^a^ The intra-day relative standard deviation of (5, 10, and 15 µg/mL) of PIO, ALO, GLM, and VLD in triplicate using the proposed method

^b^ The inter-day relative standard deviation of (5, 10, and 15 µg/mL) of PIO, ALO, GLM, and VLD in triplicate using the proposed method

**Table 2:** Assay bioanalytical validation parameters of PIO, ALO, GLM, and VLD in human plasma

Parameter	PIO	ALO	GLM	VLD
**Linearity range (µg/ mL)**	5–25	5–25	5–25	5–25
**Correlation coefficient (r)**	0.9999	0.9998	0.9997	0.9991
**Slope**	5.5162	8.4906	4.2208	12.0195
**Intercept**	1.3595	-0.6215	0.3480	-25.3309
**LOD (µg/ mL)**	0.2972	0.6271	0.9472	1.6301
**LOQ (µg/mL)**	0.9007	1.9004	2.8702	4.9398
**Accuracy (R %)**	99.77	98.66	99.37	100.87
**Precision**				
**Repeatability (RSD%)** ^**a**^ **Intermediate Precision (RSD%)** ^**b**^	0.295 0.528	0.280 0.781	0.131 1.680	0.891 1.231

^a^ The intra-day relative standard deviation of (5, 15, and 25 µg/mL) of PIO, ALO, GLM, and VLD in triplicate using the proposed method

^b^ The inter-day relative standard deviation of (5, 15, and 25 µg/mL) of PIO, ALO, GLM, and VLD in triplicate using the proposed method

#### Limit of detection (LOD) and limit of quantitation (LOQ)

LOD and LOQ were calculated and illustrated in Tables 1&2. Small detection and quantitation limit values indicate the sensitivity of the proposed MUPLC method. LOD and LOQ were determined from the standard deviation of the response and the slope.

#### Accuracy

The accuracy of the suggested method was assessed by the recovery study through the analysis of 3 different quality control concentrations of each drug (5, 10, and 15 µg/mL) for the pure form and (5, 15, and 25 µg/mL) for the spiked plasma in triplicate and calculating the mean recovery percentage (R % ±SD). The excellent recoveries indicated good accuracy of the method, Tables 1&2.

#### Precision

The repeatability of the suggested method was estimated by the analysis of three replicates of three quality control concentration levels of each drug (5, 10, and 15 µg/mL) for the pure form and (5, 15, and 25 µg/mL) for the spiked plasma on the same day. The values of precision or the relative standard deviation (RSD) were calculated. The intermediate precision was assessed by analysis of the same samples on three successive days. The obtained law RSD values were shown in Tables 1&2 which indicates that the suggested method is highly precise.

#### Stability of plasma samples

The stability of PIO, ALO, GLM, and VLD in plasma samples was investigated by triplicate analysis of the low and high-quality control samples.

##### Short-term stability

Short-term stability was checked by measuring the thawed LQC and HQC samples after being maintained at room temperature for 6 h.

##### Long-term stability

Long-term stability was assessed by analyzing LQC and HQC samples stored in the freezer at -80 °C for 14 days.

##### Freeze-thaw stability

Freeze-thaw stability of LQC and HQC samples was carried out after three freeze-thaw cycles where samples were frozen at -70 °C, and then samples were left to thaw spontaneously at room temperature and frozen again.

Stability was determined by calculating the % RSD as shown in Table 3.

**Table 3:** Stability of PIO, ALO, GLM, and VLD in human plasma

Parameter	Mean^*^ ±SD			
	PIO	ALO	GLM	VLD
**Short-term stability**	103.43 **±** 0.734	98.34 ± 1.804	100.96 ± 0.638	96.96 ± 0.896
**Long-term stability**	101.14 ± 2.134	101.77 ± 2.124	100.36 ± 1.528	98.78 ± 1.369
**Freeze-thaw stability**	101.67 ± 2.973	102.76 ± 2.524	100.43 ± 2.130	97.21 ± 2.618

^*^Mean of three determinates

LCQ sample of 5 µg/mL and HQC sample of 25 µg/mL

#### Robustness

The robustness of the proposed method was checked by measuring of resolution after deliberating changes in the experimental method parameters including pH (± 0.5), flow rate (± 0.02 min), percent of solvent A (± 2%), and solvent B (± 2%). The induced slight changes did not result in any no significant change which indicates the method is robust and reliable, (Supplementary Table 1).

#### System suitability test

A system suitability test was used to evaluate the effectiveness of the developed chromatographic separation parameters. System suitability test parameters include the resolution factor, tailing factor, theoretical plates number or column efficiency, and retention time as shown in Table 4. The obtained results complied with food and drug administration (FDA) guidance.

**Table 4:** System suitability parameters of the suggested MUPLC method

Parameter	Obtained value				Recommended value *
	PIO	ALO	GLM	VLD	
**Resolution (R**_**s**_ **)**					
**PIO** **ALO** **GLM**	…….	8.88	13.94 5.12	17.82 9.03 3.91	> 2
**Selectivity (α)**					
**PIO** **ALO** **GLM**	…….	2.25	2.99 1.33	3.56 1.58 1.19	> 1
**Tailing factor (T)**	1.15	1.05	1.32	1.02	< 2
**Theoretical plate number (N)**	2152.32	4019.47	6788.88	9376.37	> 2000
**Capacity factor (K`)**	0.82	3.11	4.46	5.50	1–10 acceptable

*Values defined by FDA Center of Drug Evaluation and Research’s reviewer guidance on validation of chromatographic methods (November 1994)

### Application to pharmaceutical tablets

The developed MUPLC method was applied for the analysis of different pharmaceutical dosage forms applying the standard addition technique. The recovery % of the studied drugs from their pharmaceutical tablets was calculated and illustrated in Table 5. No interference from excipients was observed which proved the applicability of the proposed method as shown in Figs. 2, 3, 4, 5 and 6. The results of the analysis of pharmaceutical tablets were compared to the results of the reported methods [18, 27, 43, 44] by statistical tests which showed no significant difference as shown in Table 6.

**Table 5:** Result of PIO, ALO, GLM, and VLD determination in their dosage forms by the proposed MUPLC method and application of standard addition technique

**Product**		Found* *% ± RSD*	Standard addition					
			Taken (µg/mL)	Added		Found^**^ (µg/mL)	Recovery%	
**prendaglim plus**	**PIO**	101.23 ± 1.717	3	5		4.93	98.70	
				10		10.05	100.47	
				15		15.22	101.48	
			**Mean**	100.21				
			**RSD%**	1.407				
	**ALO**	100.57 ± 1.079	5	5		4.96	99.19	
				10		10.05	100.52	
				15		15.15	100.97	
			**Mean**	100.23				
			**RSD%**	0.922				
**Zanoglide**	**PIO**	101.43 ± 1.195	15	5	5.07		101.34	
				10	10.21		102.05	
				15	15.25		101.67	
			**Mean**	101.69				
			**RSD%**	0.353				
	**GLM**	99.62 ± 1.736	2	5	5.01		100.17	
				10	9.87		98.71	
				15	14.86		99.04	
			**Mean**	99.31				
			**RSD%**	0.768				
**Prendaglim**	**ALO**	100.88 ± 1.705	5	5	5.11		102.11	
				10	9.95		99.53	
				15	15.15		100.97	
			**Mean**	100.87				
			**RSD%**	1.279				
**Gliptus**	**VLD**	101.33 ± 0.497	25	5	4.91		98.25	
				10	10.15		101.47	
				15	15.12		100.80	
			**Mean**	100.17				
			**RSD%**	1.692				

^*^Mean of five determinations

**Mean of three determinations

**Table 6:** Statistical analysis of the results obtained by applying the proposed MUPLC method and the reported method for the determination of ALO, VLD, PIO, and GLM in different pharmaceutical dosage forms

Parameter	Proposed method								
	PIO/ALO		PIO/GLM				ALO	VLD	
	PIO	ALO	PIO		GLM				
**N**	5	5	5		5		5	5	
**Mean** ^*^	101.23	100.57	101.43		99.62		100.88	101.33	
**SD**	1.738	1.085	1.212		1.729		1.761	0.504	
**Variance**	3.021	1.177	1.470		2.989		3.101	0.254	
**Student t-test (6.39)** ^**^	0.15	0.34	1.29		0.31		0.41	1.23	
**The Variance ratio F-test (2.31)** ^**^	1.22	1.91	1.56		1.10		3.06	1.48	
**Parameter**	**Reported method**								
	**PIO/ALO** ^**[18]**^			**PIO/GLM** ^**[27]**^			**ALO** ^**[43]**^		**VLD** ^**[44]**^
	**PIO**	**ALO**		**PIO**		**GLM**			
**N**	5	5		5		5	5		5
**Mean** ^*^	101.38	100.77		100.31		99.95	100.50		100.89
**SD**	1.577	0.784		1.514		1.646	1.007		0.614
**Variance**	2.486	0.615		2.291		2.709	1.015		0.377

^*^Average of five experiments

^**^Figures between parentheses represent the corresponding tabulated values of t and F at P = 0.05

HPLC method [18] (C18 column, using phosphate buffer (pH 3) and methanol (45:55 v/v) at a flow rate of 0.3 mL/min and UV detection at 280 nm)

HPLC method [27] (C18 column, using acetonitrile and ammonium acetate (pH 4.5; 20 mM) 60:40 (v/v) at a flow rate of 1.0 mL/min and UV detection at 230 nm)

HPLC method [43] (C18 column, methanol: double distilled water (80:20, v/v) at a flow rate of 1 mL/min and UV detection at 222 nm)

HPLC method [44] (C18 column, using acetonitrile:0.02 M potassium di-hydrogen Phosphate (pH 4.5) (25:75 v/v) at a flow rate of 1.0 mL/min and UV detection at 215 nm)

### Analysis of spiked human plasma

The developed MUPLC method was tried to determine each drug concentration in spiked human plasma. The results of the estimation of PIO, GLM, and VLD in spiked plasma samples were satisfactory, as shown in Table 7. No interference from the plasma matrix was observed, as shown in Figs. 2, 3, 4, 5 and 6, and the chromatogram of the blank plasma (Supplementary Fig. 1) showed that there were no interfering substances present, so no pretreatment of the plasma is required.

**Table 7:** Determination of PIO, ALO, GLM, and VLD in spiked human plasma via the suggested MUPLC method

Added (µg/mL)	PIO		ALO		GLM		VLD	
	Found^*^ (µg/mL)	**R%**	Found^*^ (µg/mL)	R%	Found^*^ (µg/mL)	R%	Found^*^ (µg/mL)	R%
**5**	5.07	101.48	5.10	101.98	5.02	100.40	5.01	100.25
**10**	10.19	101.93	9.95	99.48	9.97	99.68	10.18	101.85
**20**	19.75	98.73	19.91	99.56	19.64	98.22	19.96	99.78
**25**	24.54	98.15	24.89	99.58	24.70	98.81	25.42	101.66
**Mean ± RSD**	100.07 ± 1.906		100.15 ± 1.218		99.28 ± 0.966		100.88 ± 1.016	

* Mean of three determinations

### Greenness assessment of the procedure using the analytical eco-scale

The Analytical Eco-scale scoring was applied to assess the greenness of the proposed MUPLC technique[45]. The analytical Eco-scale is based on the ideal green analytical procedures and has a total score of 100. Each parameter of the analytical procedures is assigned penalty points if it is deviated from the conception of green analysis [46]. Different parameters along the whole analysis procedure are to be evaluated including (hazards, type and amount of reagents used, energy consumption, and waste generation and its treatment). The sum penalty points were then subtracted from 100 to obtain the Eco-scale points of the method. Table 8 showed 85 Eco-score points which indicated an excellent green MUPLC method.

**Table 8:** Assessment of the greenness of the proposed MUPLC method for the determination of PIO, ALO, GLM, and VLD utilizing the Eco-scale tool

Parameter	Signal of wards	Number of pictograms	Penalty points
**Solvent**			
Propanol	2	2	4
SDS	0	0	0
Triethylamine	2	1	2
Phosphoric acid	2	1	2
Acetonitrile	2	1	2
**Instrument**			
Energy (UPLC) [ 0.1 kWh per sample]			0
Occupational hazards (analytical process hermitization)			0
**Waste**			
(1–10 mL, passivation)			5
**Total Penalty points**			Σ15
**Analytical Eco-scale total scores** ^**a b**^			85

a Analytical Eco-Scale total score = 100- total penalty points

b If the score is > 75, it represents excellent green analysis. If the score is > 50, it represents acceptable green analysis. If the score is ˂ 50, It represents inadequate green analysis

## Conclusion

The newly developed MUPLC technique was successfully employed for the simultaneous estimation of PIO, ALO, GLM, and VLD in the pulk, different commercial dosage forms, and spiked human plasma. The proposed method has the advantages of being rapid, saving time and effort. Furthermore, this proposed method was sensitive enough to be used for the pharmacokinetic study of the studied drugs in human plasma. Moreover, the developed MUPLC method showed excellent greenness as it gains a high total score by using the eco-scale scoring tool in greenness assessment.

## Electronic supplementary material

Below is the link to the electronic supplementary material.

Supplementary Material 1

Supplementary Material 2

## Data Availability

All data generated or analyzed during this study are included in this published article.
