# Supplementary figures and images for: Earth-friendly micellar UPLC technique for determination of four hypoglycemic drugs in different pharmaceutical dosage forms and spiked human plasma
Source: BMC Chem. 2023 Jul 12;17(1):74. doi: 10.1186/s13065-023-00983-6 (PMC10339510; doi:10.1186/s13065-023-00983-6)

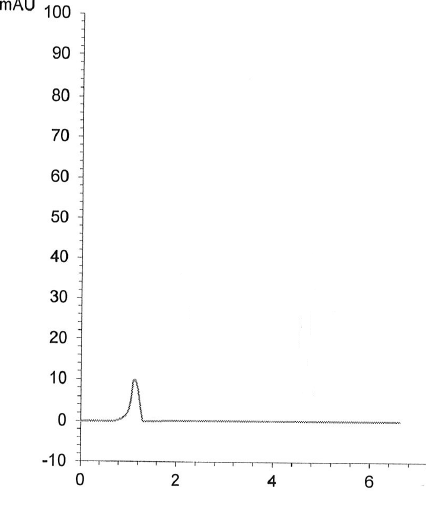


**Supplementary Figure 1. The MUPLC chromatograms of the blank plasma.**

Supplement: Supplementary file 1 — Supplementary Material 1 [file 13065_2023_983_MOESM1_ESM.docx]
