# Supplementary material for: Earth-friendly micellar UPLC technique for determination of four hypoglycemic drugs in different pharmaceutical dosage forms and spiked human plasma
Source: BMC Chem. 2023 Jul 12;17(1):74. doi: 10.1186/s13065-023-00983-6 (PMC10339510; doi:10.1186/s13065-023-00983-6)
Supplement: Supplementary file 2 — Supplementary Material 2 [file 13065_2023_983_MOESM2_ESM.docx]

**Supplementary Table (1): Robustness of the proposed MUPLC method for separation of PIO, ALO, GLM, and VLD.**

| **Parameter** | | **Resolution (R_S_)** | | | | | |
| --- | --- | --- | --- | --- | --- | --- | --- |
|  |  | **ALO from PIO** | **GLM from PIO** | **GLM from ALO** | **VLD from**  **PIO** | **VLD from ALO** | **VLD from GLM** |
| **Solvent A** | **83%** | **8.67** | **13.41** |  | **17.40** |  | **3.80** |
|  | **85%** | **8.88** | **13.94** | **5.12** | **17.82** | **9.03** | **3.91** |
|  | **87%** | **8.72** | **13.56** |  | **17.54** |  | **3.94** |
|  | **RSD%** | **1.253** | **2.003** |  | **1.216** |  | **1.898** |
| **Flow rate** | **0.18** | **8.75** | **13.56** |  | **17.51** |  | **3.86** |
|  | **0.20** | **8.88** | **13.94** | **5.12** | **17.82** | **9.03** | **3.91** |
|  | **0.22** | **8.90** | **13.75** |  | **17.74** |  | **4.00** |
|  | **RSD%** | **0.921** | **0.014** |  | **0.910** |  | **1.808** |
| **pH** | **5.50** | **8.71** | **13.50** |  | **17.58** |  | **3.91** |
|  | **6.00** | **8.88** | **13.94** | **5.12** | **17.82** | **9.03** | **3.91** |
|  | **6.50** | **8.75** | **13.67** |  | **17.75** |  | **4.05** |
|  | **RSD%** | **1.012** | **1.619** |  | **0.697** |  | **2.043** |
| **Solvent B** | **13%** | **8.86** | **14.04** |  | **17.75** |  | **4.00** |
|  | **15%** | **8.88** | **13.94** | **5.12** | **17.82** | **9.03** | **3.91** |
|  | **17%** | **8.97** | **13.66** |  | **17.29** |  | **3.90** |
|  | **RSD%** | **0.658** | **1.413** |  | **1.634** |  | **1.399** |
